# Safety Profile of Liver FibroScan in Patients with Cardiac Pacemakers or Implantable Cardioverter-Defibrillators

**DOI:** 10.1155/2017/7298032

**Published:** 2017-02-27

**Authors:** Yin Chan, Stephanie Pranke, Farid Rashidi, Shravan Nosib, Lawrence Worobetz

**Affiliations:** ^1^Division of Gastroenterology, Department of Medicine, University of Saskatchewan, Saskatoon, SK, Canada; ^2^Department of Medical Imaging, University of Saskatchewan, Saskatoon, SK, Canada; ^3^Division of Cardiology, Department of Medicine, University of Saskatchewan, Saskatoon, SK, Canada

## Abstract

*Background*. Emerging evidence suggests that nonalcoholic fatty liver disease (NAFLD) is associated with coronary artery diseases and arrhythmias. The FibroScan (Echosens, France), a widely available, noninvasive device, is able to detect liver fibrosis and steatosis within this patient population. However, the FibroScan is currently contraindicated in patients with cardiac pacemakers (PM) or implantable cardioverter-defibrillators (ICD).* Objective*. To determine the safety profile of FibroScan testing in patients with PM or ICD.* Methods*. Consecutive outpatients undergoing routine device interrogations at a tertiary level teaching hospital underwent simultaneous liver stiffness measurements. PM or ICD performance data, device types, patient demographics, medical history, and previous laboratory and conventional liver imaging results were collected.* Results*. Analysis of 107 subjects with 33 different types of implanted cardiac devices, from 5 different companies (Medtronic, Sorin, ELA Medical, Boston Scientific, and St. Jude), did not demonstrate any adverse events as defined by abnormal device sensing/pacing or ICD firing. This population included high risk subjects undergoing active pacing (*n* = 53) and with right pectoral PM placement (*n* = 1). None of the subjects had any clinical signs of decompensated congestive heart failure or cirrhosis during the exam.* Conclusion*. TE with FibroScan can be safely performed in patients with PM or ICD.

## 1. Introduction

Assessment of liver fibrosis in patients with chronic liver diseases provides staging and prognostic information critical in establishing treatment priorities. The gold standard evaluation of liver fibrosis is hampered by the invasive nature of liver biopsies [1]. The use of noninvasive tests to estimate the degree of liver fibrosis and thereby disease severity is now well established globally [2].

Ultrasound-based liver stiffness measurement (LSM) using transient elastography is in wide clinical use both in Canada and internationally. One common point-of-care device is the FibroScan (Echosens, France), which has been extensively validated in a range of clinical applications including patients with chronic hepatitis C, chronic hepatitis B, alcoholic liver disease, and nonalcoholic fatty liver disease (NAFLD) [3].

FibroScan was licensed for use in Canada in 2009 for liver diagnosis. Currently there are no absolute contraindications according to Health Canada or in other jurisdictions where it is approved. However, to avoid unknown risks of potential interaction, the manufacturer has advised against the use of the device in pregnant women and in patients with active implantable medical device [4]. Review of the literature showed that these two populations have been specifically excluded in all previously reported studies.

The precaution regarding the use of FibroScan in subjects with cardiac pacemaker (PM) or implantable cardioverter-defibrillators (ICD) is puzzling. It is unclear if the original warning was prompted by the vibrational or ultrasound component of the technology. Reviews of the literature have not demonstrated any known interaction between medical ultrasound technology and implanted cardiac device. In fact, cardiac ultrasound or echocardiogram is routinely performed in this patient population. Other manufacturers of ultrasound-based devices utilizing the same principle of transient elastography found in the FibroScan have not issued similar warnings [5, 6]. These other devices have been used worldwide for clinical applications including breast, thyroid, and prostate.

More than 200,000 Canadians are estimated to be living with PM or ICD [7]. The prevalence of liver disease in this population is not known. Cardiac hepatopathy and amiodarone-induced liver disease are unique risk factors in patients with cardiac disease [8, 9]. Conversely, emerging evidence suggests that NAFLD is associated with coronary artery diseases and arrhythmias [10].

Our objective was to determine the safety profile of FibroScan testing in patients with PM or ICD. Since previous clinical studies have specifically excluded this population, this was a unique opportunity to estimate the prevalence of clinically significant liver disease in adult outpatients with implantable cardiac devices.

## 2. Methods

### 2.1. Study Population

Consecutive adult (>16 years of age) outpatients undergoing routine pacemaker interrogation at the Royal University Hospital pacemaker clinic were invited to participate in the study over 8 randomly selected weeks between May 2015 and May 2016. Subjects who were pregnant, unable to tolerate lying flat, in decompensated congestive heart failure (CHF), or with other noncardiac implantable medical devices were excluded during the screening process on the date of their appointment. The Research Ethics Board at the University of Saskatchewan (Saskatoon, Saskatchewan) approved the research protocol. This trial is registered with ClinicalTrials.gov ID NCT02415348.

### 2.2. Cardiac Device Measurement

After routine cardiac device interrogation, an operator blinded to the medical history of the subject performed LSM. Throughout the scan, the cardiac device remained connected to external monitoring with supervision by the cardiac device technologist. Changes in pacemaker sensing or pacing threshold as well as lead impedance were recorded. For ICD, additional adverse outcomes included erroneous ventricular tachycardia (VT)/ventricular fibrillation (VF) detection with or without therapy delivery.

### 2.3. FibroScan Measurement

Two manufacturer-certified operators performed all LSM using FibroScan as previously described [11]. Briefly, with the patient lying supine, the tip of the transducer was placed on the skin between the ribs over the right lobe of the liver. The M probe was used (73%), unless the XL probe was specifically recommended by the onboard software. At least 10 validated measurements with a ratio of interquartile range (IQR) and median LSM of less than or equal to 30 percent were required for an interpretation of significant fibrosis or cirrhosis [12]. In the event of scan failure, a second scan was attempted in the seated upright position (5%). Scan failure was defined as inability to obtain 10 validated readings.

### 2.4. Clinical Data

At the completion of LSM, patient demographics, body mass index (BMI), ethnicity, and past cardiac and liver related medical history were obtained. Results from previous liver imaging studies (ultrasound (U/S), computed tomography (CT), and magnetic resonance imaging (MRI)), liver function tests, total bilirubin levels, and platelet counts were also collected, if available.

### 2.5. Statistical Analyses

Descriptive statistics were calculated for all applicable variables. Between-group comparisons were made using Mann–Whitney tests. A two-sided *P* < 0.05 was considered to be statistically significant. Prism 7.00 (GraphPad, USA) was used for all statistical analyses.

## 3. Results

### 3.1. Cohort Characteristics

In the 8-week study period, 415 subjects were approached for the study. Due to a lower recruitment rate (27%) than anticipated in the original study design, a total of 113 subjects out of the planned 200 were eventually recruited. Unreliable readings or failed LSM were obtained in 6 (5%) recruited subjects. The characteristics of the remaining 107 subjects that comprised the final study cohort are summarized in Table 1. Sixty-six percent of the subjects were male, while the median age and BMI were 80 years and 27 kg/m^2^, respectively. One (1%) subject had a documented history of cirrhosis on their medical record. The majority (88%) had a PM only, while the remaining 14 subjects also had ICD. Subgroups at potentially higher risk of interference included 53 (47%) subjects who were pacer dependent and the 1 (1%) subject with a right pectoral implantation site. The predominate indications for the cardiac devices were sinus node dysfunction (49%) and acquired atrioventricular node dysfunction (32%).

### 3.2. Cardiac Device Function during FibroScan

A total of 33 different types of implanted cardiac devices from 5 companies (Medtronic, Sorin, ELA Medical, Boston Scientific, and St. Jude) were tested (Table 2). The duration of LSM ranged from 5 to 20 min. During LSM, abnormality in pacemaker sensing/pacing threshold or lead impedance was not observed in any subject. For patients with ICD, no erroneous ventricular tachycardia (VT)/ventricular fibrillation (VF) or therapy delivery was detected. No adjustments to cardiac devices setting were required in any of the subjects subsequent to LSM. Furthermore, LSM was well tolerated with no significant discomfort reported.

### 3.3. Detection of Significant Fibrosis in Cohort

**Figure 1:**
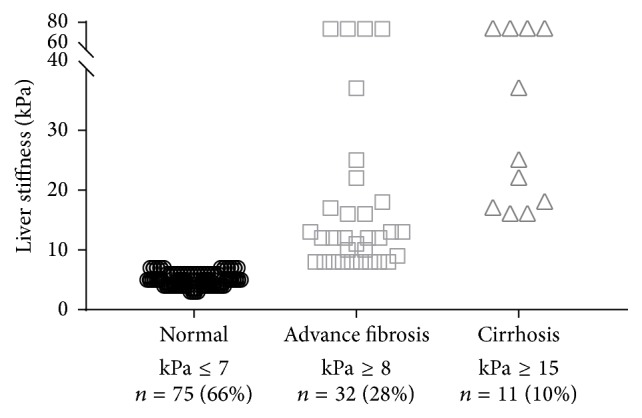
Number and percentage of subjects with normal, advanced fibrosis, and cirrhosis based on their liver stiffness measurement.

LSM thresholds for advanced fibrosis (METAVIR F2–F4) and cirrhosis (F4) have not been validated for screening in the general population. For the purpose of this study, advanced fibrosis was defined as subjects with LSM ≥8 kPa and cirrhosis in subject with LSM ≥15 kPa. Using these cutoffs, the incidences of advanced fibrosis and cirrhosis were 28% (*n* = 32) and 10% (*n* = 11), respectively. The distribution of LSM values in each group is shown in Figure 1.

**Figure 2:**
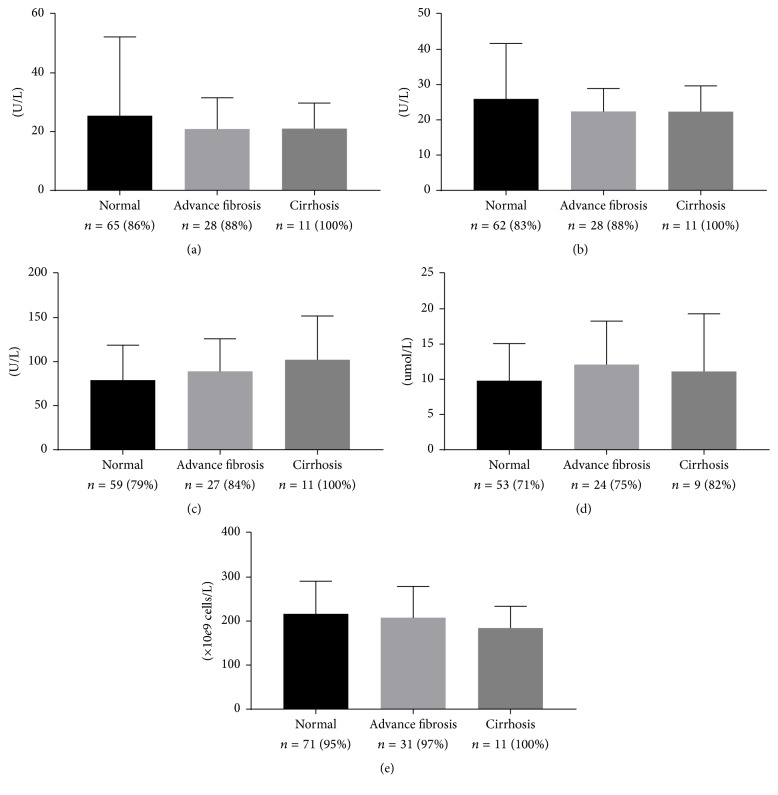
In subjects *n* (% of group) with previous laboratory investigations available, no significant difference in (a) alanine aminotransferase, (b) alanine aspartate aminotransferase, (c) alkaline phosphatase, (d) total bilirubin, or (e) platelet count was detected in subjects with elevated liver stiffness measurement (cirrhosis versus normal, Mann–Whitney tests, *P* > 0.05).

To look for laboratory evidence of liver dysfunction, incidental blood work from the preceding 5 years was analyzed. At least 71% of subjects in each group had a previous platelet count, alanine aminotransferase (ALT), alanine aspartate aminotransferase (AST), alkaline phosphatase (ALP), or total bilirubin on record. No significant difference in laboratory values was found in either the advanced fibrosis or cirrhosis group as compared to subjects with normal LSM (Figure 2). In contrast, subjects in the advanced fibrosis and cirrhosis group were more likely to have previous CT/US/MRI results suggestive of cirrhotic changes as compared to normal subjects; however the low rate of incidental liver imaging prevented meaningful statistical analysis (Table 3).

## 4. Discussion

The use of FibroScan in patients with implanted cardiac devices has been controversial. No safety issues or adverse events relating to the device have been reported in the literature since its introduction in 2003 [13]. While published trials have specifically excluded this population, it is unclear if all institutions have adopted this contraindication in the clinical setting. Certainly, there are anecdotal accounts of accidental use of FibroScan in patients with PM in our institution with no ill effect. The present study confirmed that FibroScan can be safely used in patients with PM or ICD. The sample size was limited by the time and resource needed to keep subjects under monitoring by a cardiac device technologist, as well as the low study consent rate due to the clear manufacturer contraindication. Nevertheless, given the lack of any theoretical basis for interaction and the detailed monitoring of the cardiac device during LSM, any potential risk may be far exceeded by the clinical benefit of the scan. Potential clinical benefits include secondary confirmatory testing and hepatology follow-up in a population that largely had no previous diagnosis of advanced fibrosis.

The incidence (10%) of cirrhosis in our predominately geriatric, Caucasian cohort was higher than previous reports from consecutive autopsies (3.8–4.5%) and LSM in healthy general population over 45 years of age (0.7%) [14–16]. We were unable to validate our LSM results with concurrent liver biopsies. The exception is a subject with previous biopsy proven amiodarone-related cirrhosis that was correctly detected by LSM. Other known LSM confounders applicable to this study include nonfasting subjects and lack of concurrent blood work to rule out liver inflammation or extrahepatic cholestasis in the study protocol [17–19]. Elevation of central venous pressure in CHF patients is associated with increased LSM by FibroScan and it is unclear if LSM remain persistently elevated in clinically compensated CHF [20]. Unfortunately, we were unable to collect sufficient high-quality echocardiogram results with reliable right ventricle diastolic pressure, right atrial pressure, and central venous pressure to rule out the effect of venous congestion on our LSM measurements [21, 22]. Despite these limitations, retrospective review of incidental liver imaging supports the hypothesis that a proportion of subjects with cirrhotic range LSM in our study did indeed have compensated disease. Future large cohort studies with biopsy validation are required to estimate the true prevalence of cirrhosis in patients with implanted cardiac devices.

Subjects with abnormal FibroScan results were invited for further hepatology workup at the conclusion of the study. Until these follow-up studies are completed, it is unclear if amiodarone-induced liver disease and cardiac hepatopathy are significant contributors to the prevalence of chronic liver disease in this population.

In summary, we have demonstrated that LSM by FibroScan can be safely used in patients with PM or ICD. While only 33 different types of devices were tested, the similarity in the underlying technology should ensure a similar risk profile for this broad class of medical devices. These patients may also carry a higher risk of developing cirrhosis, but further validation studies are required.

## Figures and Tables

**Table 1:** Baseline characteristics.

Characteristics	All subjects (*n* = 107)
Demographic	
** **Age, years	80 (71–86)
** **Male sex	66 (58%)
** **Body mass index, kg/m^2^	27 (24–30)
** **Ethnicity, Caucasian	109 (96%)
** **Known cirrhotic	1 (1%)
Cardiac device history	
** **Pacemaker	99 (88%)
** **Implantable cardioverter-defibrillators	14 (12%)
** **Years after implantation	4 (2–7)
** **Pacer-dependent	53 (47%)
** **Right pectoral implantation site	1 (1%)
Cardiac device indication	
** **Sinus node dysfunction	55 (49%)
** **Acquired AV block	36 (32%)
** **DCM or VT/VF	12 (11%)

Data presented as *n* (%) or median (interquartile range). AV: atrioventricular node dysfunction. DCM: dilated cardiomyopathy. VT: ventricular tachycardia. VF: ventricular fibrillation.

**Table 2:** Type and number of cardiac devices tested.

Device manufacturer	Device name	Subjects (*n*)
Boston Scientific	Insignia I Plus 1194	1

ELA Medical	Symphony DR 2250	2
	Symphony DR 2550	8
	Symphony SR 2250	2
	Symphony SR 2550	1

Medtronic	Adapta ADDR01	1
	Adapta ADDR03	9
	Adapta ADSR03	5
	Adapta ADVDD01	1
	Adapta L ADDRL1	3
	Consulta CRT-D D234TRK	1
	Protecta XT CRT-D D354TRG	1
	Protecta XT DR D354DRG	3
	Protecta XT DR D354DRM	1
	Protecta XT VR D354VRG	3
	Protecta XT VR D354VRM	1
	Sensia L SEDRL1	1
	Virtuoso DR D164AWG	2

Sorin	Reply 200 DR W2.151	2
	Reply 200 SR W2.151	1
	Reply DR W1.52	3
	Reply DR W2.74	7
	Reply DR W2.92	21
	Reply SR W1.52	2
	Reply SR W2.74	5
	Reply SR W2.92	6
	Symphony DR 2550	1

St. Jude Medical	Accent DR 2112	2
	Accent SR 1110	2
	Assurity DR 2260	6
	Ellipse VR 1277-36Q	1
	Endurity 1160	1
	Promote RF CRT-D 3213-36	1

**Table 3:** Correlation of FibroScan results with retrospective, incidental liver imaging studies.

	Normal	Advanced fibrosis	Cirrhosis
Total subjects (*n*)	75	32	11
Previous liver imaging available (*n*, %)	26 (35%)	15 (47%)	7 (64%)
Radiographic cirrhosis (*n*, %)	0 (0%)	5 (16%)	4 (57%)
